# The Effects of Age and Dose on Gene Expression and Segmental Bone Defect Repair After BMP‐2 Delivery

**DOI:** 10.1002/jbm4.10068

**Published:** 2018-07-16

**Authors:** Albert Cheng, Laxminarayanan Krishnan, Lisa Tran, Hazel Y Stevens, Boao Xia, Nari Lee, Joseph K Williams, Greg Gibson, Robert E Guldberg

**Affiliations:** ^1^ George W. Woodruff School of Mechanical Engineering Georgia Institute of Technology Atlanta GA USA; ^2^ Parker H. Petit Institute for Bioengineering and Bioscience Georgia Institute of Technology Atlanta GA USA; ^3^ Emory University School of Medicine Atlanta GA USA; ^4^ Emory University Pediatric Engineering Research Summer Experience Atlanta GA USA; ^5^ Children's Healthcare of Atlanta Atlanta GA USA; ^6^ Center for Integrative Genomics School of Biological Sciences Georgia Institute of Technology Atlanta GA USA

**Keywords:** AGING, BMPS, INJURY/FRACTURE HEALING, PRECLINICAL STUDIES, BONE QCT/μCT

## Abstract

Age is a well‐known influential factor in bone healing, with younger patients generally healing bone fractures more rapidly and suffering fewer complications compared with older patients. Yet the impact age has on the response to current bone healing treatments, such as delivery of bone morphogenetic protein 2 (BMP‐2), remains poorly characterized. It remains unclear how or if therapeutic dosing of BMP‐2 should be modified to account for age‐related differences in order to minimize potential adverse effects and consequently improve patient bone‐healing outcomes. For this study, we sought to address this issue by using a preclinical critically sized segmental bone defect model in rats to investigate age‐related differences in bone repair after delivery of BMP‐2 in a collagen sponge, the current clinical standard. Femoral defects were created in young (7‐week‐old) and adult (8‐month‐old) rats, and healing was assessed using gene expression analyses, longitudinal radiography, ex vivo micro‐computed tomography (µCT), as well as torsional testing. We found that young rats demonstrated elevated expression of genes related to osteogenesis, chondrogenesis, and matrix remodeling at the early 1‐week time point compared with adult rats. These early gene expression differences may have impacted long‐term healing as the regenerated bones of young rats exhibited higher bone mineral densities compared with those of adult rats after 12 weeks. Furthermore, the young rats demonstrated significantly more bone formation and increased mechanical strength when BMP‐2 dose was increased from 1 µg to 10 µg, a finding not observed in adult rats. Overall, these results indicate there are age‐related differences in BMP‐2‐mediated bone regeneration, including relative dose sensitivity, suggesting that age is an important consideration when implementing a BMP‐2 treatment strategy. © 2018 The Authors *JBMR Plus* published by Wiley Periodicals, Inc. on behalf of American Society for Bone and Mineral Research.

## Introduction

The repair of large bone defects represents one of the most challenging problems faced by orthopedic and craniofacial surgeons today. The gold standard in treatment is autograft,^1^ but graft substitutes and/or delivery of osteoinductive proteins such as the bone morphogenetic protein (BMP) family are also common alternatives with demonstrated efficacy in bone regeneration.^2^, ^3^, ^4^ Although the effects of age on endogenous bone healing have been well documented, both in preclinical animal models^5^, ^6^, ^7^, ^8^, ^9^, ^10^ and human patients,^11^, ^12^, ^13^ the influence of age on the response to current bone healing treatments is much less informed. It has been demonstrated in vitro that age (as well as donor site) influences the responsiveness of bone marrow stromal cells to BMP‐2.^14^ Indeed, clinical studies have shown similar trends, as use of BMP‐2 and allograft in spine fusion in elderly patients (>65 years) demonstrated lower fusion rate and longer fusion time compared with younger patients (<65 years) receiving the same treatment.^15^ Yamaji and colleagues also showed in vivo that the thickness of new bone formed when BMP‐2 is injected into a palatal site was much lower in elderly 70‐week‐old rats compared with young 10‐week‐old rats.^16^ Importantly, this study also demonstrated an age‐related difference in the osteoinductive response to increasing doses of BMP‐2. Whereas the elderly animals showed an increase in thickness of new bone over a large range of increasing BMP‐2 doses (0 to 16 μg), the younger animals exhibited a much smaller benefit window (0 to 4 μg), beyond which a significant reduction in new bone thickness was actually observed. Taken together, these results indicate that BMP‐2 dose sensitivity is influenced by age, and the optimal dose requirement for young animals/patients is likely to be lower than that for older animals/patients.

And yet, to the best of our knowledge, clinicians rarely take into account patient age when determining the dose of BMP‐2 to be delivered. This is problematic because it has been well documented that delivery of supraphysiological doses of BMP‐2 is fraught with risks, including heterotopic mineralization, osteolysis, infection, and potentially life‐threatening inflammation/swelling.^17^, ^18^, ^19^ Furthermore, use of higher BMP‐2 doses has been shown to result in heterotopic ossification and formation of abnormal spongy bone with thin trabecular networks.^20^, ^21^ These complications could potentially be most devastating in the pediatric population, with reports of massive inflammatory reactions and acute pain in some pediatric patients.^22^, ^23^, ^24^ As a result, the use of any BMPs in skeletally immature patients is currently not approved by the U.S. Food and Drug Administration (FDA).^25^ Despite these known risks, off‐label use of BMP‐2 still occurs due to a lack of better alternatives in many scenarios, as well as documented successful cases without any noticeable complications.^24^, ^26^, ^27^ In the absence of controlled trials to optimize therapeutic dosing (which remain unfeasible because of ethical considerations and costs), the use of relevant preclinical animal models represent the best approach to gain insight into this important question of how age influences the response to BMP‐2 treatment during bone healing.

We have previously established a critically sized segmental bone defect model in rats^28^ to study bone regeneration and, furthermore, have shown this model to be robust enough to qualitatively and quantitatively evaluate the efficacy of different treatments, including autograft and the clinical standard BMP‐2 in collagen sponge.^4^, ^29^, ^30^ Here, we adapted this model to 7‐week‐old young adolescent (young) and 8‐month‐old middle‐aged adult (adult) rats in order to investigate how age impacts BMP‐2‐mediated bone repair at both a subhealing (historically does not result in bridging) low dose (1 µg) and relatively higher dose (10 µg) of BMP‐2.^29^ We hypothesized that the osteogenic response would be a function of animal age, with young animals showing greater bone regeneration at the low dose and signs of supraphysiological levels of BMP‐2 at the high dose, and the adult animals healing only at the high dose. We further hypothesized that young rats would demonstrate elevated early expression of genes related to bone healing as well as inflammation (particularly at the high BMP‐2 dose), and this would correlate to improved long‐term bone regeneration compared with the adult rats. Early (1‐week post‐surgery) gene expression profiles of young and adult animals at the two doses of BMP‐2 were assessed by quantitative real‐time polymerase chain reaction (qRT‐PCR) to identify potential mechanistic differences. To compare bone regeneration, defect mineralization was monitored over time using longitudinal radiographs and end point micro‐computed tomography (µCT) scans were analyzed for quantitative measures of new bone formation. Finally, the biomechanical properties of the regenerated femurs were measured by torsional testing, serving as a functional metric to assess healing, while routine histology of harvested femora provided qualitative assessment of the newly formed bone.

## Materials and Methods

### Animals

For these studies, 7‐week‐old and 8‐month‐old (retired breeders) male Sprague Dawley rats (Harlan Laboratories/Envigo, Indianapolis, IN, USA) were used. Herein age refers to age at the time of surgery. Rats were pair housed in individually ventilated caging (Tecniplast, West Chester, PA, USA) with a tunnel and gnawing blocks (Bio‐Serv, Prospect, CT, USA) for enrichment. Bedding was a mixture of corn cob and processed paper. Purina Mills International #5001 was fed *ad libitum*. Filtered tap water treated with ultraviolet light was provided *ad libitum* in bottles. Sentinel results from Charles River Laboratories (Wilmington, MA, USA) International Rat Prevalent PRIA testing were negative for all pathogens in the housing room. All animals were allowed to acclimate for at least 2 weeks before any procedures were performed. After each procedure, a divider was temporarily placed in the cage for better monitoring of postoperative recovery. Animals were randomly allocated to treatment groups.

### Surgical procedure

All surgical procedures were approved by the Georgia Institute of Technology Institutional Animal Care and Use Committee. The surgical procedure has been described previously.^28^ Anesthesia was induced and maintained using isoflurane (Henry Schein Animal Health, Dublin, OH, USA) inhalation. Briefly, an anterolateral skin incision was made in the thigh followed by blunt dissection to separate the overlaying muscles to reach the femur bone. Limited extension of this muscle window allowed for placement of a radiolucent polysulfone fixation plate for internal stabilization. Critically sized 8‐mm defects were created in the mid‐diaphysis of the femur using an oscillating saw. A collagen sponge loaded with BMP‐2 was then delivered to the defect site, and finally the muscle and skin were closed using 4‐0 vicryl suture and wound clips, respectively. Before surgery, all animals were given a subcutaneous injection of sustained‐release buprenorphine (ZooPharm, Windsor, CO, USA) for analgesia.

### Collagen sponge preparation and insertion

The day before each surgery, BMP‐2 (Pfizer, New York, NY, USA) in 0.1% rat serum albumin (Sigma‐Aldrich, St. Louis, MO, USA) in 4 mM HCl solution was prepared and stored overnight at 4°C. Collagen sponge cylinders ∼5 mm in diameter and 10 mm in length were created by biopsy punch from a sheet of collagen sponge (Kensey Nash/DSM, Exton, PA, USA). All collagen sponges were used directly from the manufacturer's sterile packaging or sterilized by ethylene oxide if previously opened. During the surgery, the collagen sponge cylinders were transferred to a 24‐well plate, and then 150 µL of the BMP‐2 solution was carefully loaded onto each cylinder. The sponge was left for ∼10 minutes to soak up any residual BMP‐2 solution in the well before being gently press‐fit into the bone defect.

### qRT‐PCR and analyses

At 1 week post‐surgery, all tissue within the bone defect as well as a portion of the surrounding muscle were harvested and stored separately in RNAlater solution (Thermo Fisher Scientific, Waltham, MA, USA) at 4°C. Bone and muscle tissue were similarly harvested from naïve age‐matched animals for both age groups. Within 1 month of harvesting tissues, RNA was isolated by following the QIAzol extraction method (Qiagen, Valencia, CA, USA). RNA quality and concentration were assessed using the 2100 Bioanalyzer system (Agilent, Santa Clara, CA, USA) and Nanodrop ND‐1000 Spectrophotometer (Thermo Fisher Scientific), respectively. Subsequently, 300 ng of RNA from each sample was converted to DNA using the RT2 First Strand cDNA kit (Qiagen) and then stored at −20°C (all remaining RNA was stored at −80°C). Samples and TaqMan primers (Thermo Fisher Scientific; see Supplemental Fig. S1 for more detailed primer information) for 46 genes of interest were prepared for loading into 48.48 gene expression IFC chips (Fluidigm, South San Francisco, CA, USA) according to the manufacturer's protocols.^31^ The chips were then primed, loaded with sample and primer reaction mixes, and then run through the BioMark System (Fluidigm). AccuRef rat universal cDNA (Gene Scientific, Rockville, MD, USA) and distilled water were used as positive and negative controls, respectively, to ensure run fidelity.

Auto‐estimation of threshold cycle (Ct) was performed within the native Fluidigm software and the data exported for further processing. Variances of five housekeeping genes (*Hprt1, Rplp1, Rpl13a, Gapdh*, and *Ppia*) were examined, and the three housekeeping genes with the lowest variance over all the runs were chosen for normalization (*Gapdh* and *Ppia* were excluded). Relative expression of genes of interest was determined by normalizing to the average of the three chosen housekeeping genes (Ct^Gene^ − Ct ^Arithmetic Mean of 3 HK^) for each sample.^32^ Finally, the normalized expression values were inverted by subtracting from 31 (1 greater than the highest observed cycle number), which allowed for more straightforward analysis—higher adjusted Ct numbers represent higher relative expression levels. These data were then imported into JMP Genomics 8 software (SAS Institute Inc, Cary, NC, USA) and analyzed using the basic expression workflow module. Principal variance component analysis (PVCA) was performed to determine the proportion of variance for each of the major principal components explained by age, BMP‐2 dose, and their interaction components, and the principal components were examined visually to assess the degree of sample clustering on scatter plots. Hierarchical clustering analysis allowed grouping of samples in an unbiased manner according to similarity in expression profiles. Subsequently, significant differences in gene expression for this data set of 38 samples and 46 genes in our array were assessed using ANOVA and gene‐specific linear modeling,^33^ controlling for multiple comparisons with a 5% Benjamini‐Hochberg false discovery rate (FDR). The sample sizes for each group were: Young, unoperated (*n* = 7), Young, 1 µg BMP‐2 (*n* = 8), Young, 10 µg BMP‐2 (*n* = 7), Adult, unoperated (*n* = 6), Adult, 1 µg BMP‐2 (*n* = 5), Adult, 10 µg BMP‐2 (*n* = 5).

### Radiography and micro‐computed tomography

To qualitatively assess longitudinal bone regeneration, 2D in vivo digital radiographs were acquired with an MX‐20 digital machine (Faxitron X‐ray Corp, Tucson, AZ, USA) at 2, 4, 8, and 12 weeks post‐surgery. Bridging scores were assigned to each radiograph by two blinded investigators where bridging was defined as continuous bone spanning the entire defect space (from bone end to bone end). In instances of disagreement, a third blinded investigator served as tiebreaker. New bone formation was quantitatively evaluated using 3D µCT at 12 weeks post‐surgery. Ex vivo scans of the harvested femora were performed before mechanical testing using the vivaCT40 (Scanco Medical, Bruttisellen, Switzerland) at a 21 µm voxel size, 55 kVp voltage, and a 145 µA current. A threshold corresponding to 50% of native cortical bone density was applied to segment bone mineral and identify newly regenerated bone, as established previously;^28^ these thresholds were determined to be ∼315.7 and 406.6 mg hydroxyapatite/cm^3^ for the young and adult animals, respectively. The volume of interest (VOI) consisted of the central 5.54 mm (264 slices) of the 8 mm defect. Mineralization was further defined as orthotopic (defect bone volume), within a 6 mm circular contour, which corresponded to the diameter of intact bone, or heterotopic (ectopic bone volume), which formed outside of the 6 mm circular contour, as established previously.^20^, ^34^ All µCT and biomechanical test data were normalized to native bone values (unoperated contralateral femur) to account for normal growth‐related changes with age and animal‐to‐animal variability. The sample sizes for each group were: Young, 1 µg BMP‐2 (*n* = 9), Young, 10 µg BMP‐2 (*n* = 12), Adult, 1 µg BMP‐2 (*n* = 10), Adult, 10 µg BMP‐2 (*n* = 6).

### Biomechanical testing

Torsional testing to failure was performed as previously described.^28^ Femurs were excised at 12 weeks post‐surgery, wrapped in PBS‐soaked gauze, and stored at −20°C until testing could be performed. On the day of testing, samples were thawed, the surrounding soft tissues were excised, and the femora were first μCT scanned, as described above. Subsequently, the fixation plate was removed so that the native bone ends could be potted in Wood's metal (Alfa Aesar, Haverhill, MA, USA). The potted femurs were tested to failure in torsion at a rotation rate of 3° per second using the EnduraTEC ELF3200 axial/torsion testing system (TA Instruments, New Castle, DE, USA). Failure strength was determined by locating the peak torque within the first 60° of rotation. Torsional stiffness was calculated by finding the slope of the linear region before failure in the torque‐rotation plot. The sample sizes for each group were: Young, 1 µg BMP‐2 (*n* = 9), Young, 10 µg BMP‐2 (*n* = 12), Adult, 1 µg BMP‐2 (*n* = 10), Adult, 10 µg BMP‐2 (*n* = 6).

### Histological analyses

Two representative samples from each treatment group were harvested post‐mechanical testing and fixed in 10% neutral‐buffered formalin at room temperature for 48 hours. Samples were then switched to PBS and sent to HistoTox Labs (Boulder, CO, USA) for decalcification, processing, and hematoxylin and eosin (H&E) and Safranin‐O staining. Picrosirius red staining was performed in‐house according to established protocols. Immunohistochemistry of inducible nitric oxide synthase (iNOS) expression was completed using an anti‐rat antibody (Abcam, Cambridge, MA, USA) following heat‐mediated antigen retrieval at 60°C overnight in sodium citrate buffer solution (pH 6.0). All images were taken in the middle of the bone defect.

### Statistical analyses and power calculation

All data are reported as mean ± standard deviation. Unless otherwise noted, significance was determined using 1‐way analysis of variance (ANOVA) with multiple comparisons made by Tukey's post hoc test. In cases of unequal variances (according to the Brown‐Forsythe test), the nonparametric equivalent Kruskal‐Wallis test with Dunn's post hoc test was used instead. Significance was determined by a *p* value < 0.05. All statistical calculations were performed using GraphPad Prism 7 software (GraphPad, La Jolla, CA, USA). Sample sizes were determined by performing a power analysis in G*Power software based on bone volume and maximum torque results obtained from previous studies. These power calculations, along with historical data using this segmental bone defect rat model, suggest a sample size of 7 to 8 is sufficient to give statistical differences between groups.

## Results

### Early gene expression characterization in regenerating bone

**Figure 1:**
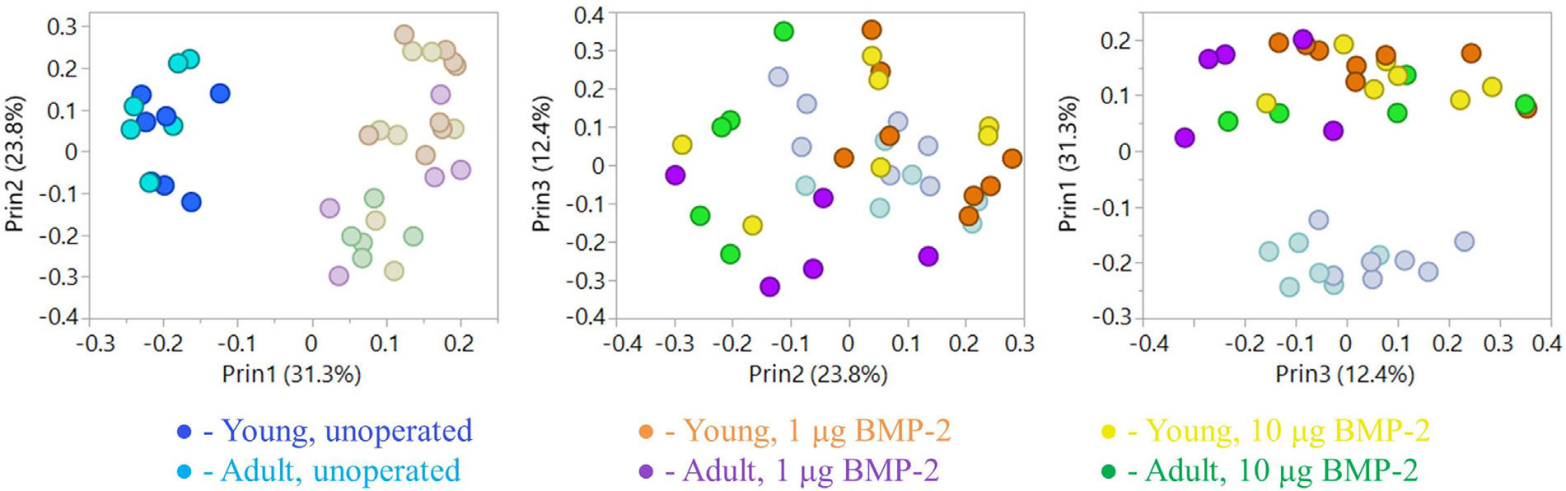
Principal component analysis (PCA) of bone defect gene expression at 1 week. PCA scatter plots revealed sample segregation across multiple principal components. The PC1 axis exhibited distinct separation between unoperated controls (which are highlighted in the left panel) from treated samples. Furthermore, among the treated samples, there was separation based on age along PC2 (irrespective of BMP‐2 dose), as can be seen in the middle panel with the adult (purple and green) and young (orange and yellow) samples highlighted. Finally, the panel on the right indicates slight separation based on dose along PC3 between the l µg (orange and purple) and 10 µg (yellow and green) BMP‐2 samples.

Local gene expression of the regenerating bone defect tissue at 1 week post‐surgery was assessed across a panel of 46 genes (Table 1). Pairwise scatter plots of the first 3 principal components (Fig. 1) demonstrated clear separation of unoperated controls from operated/treated groups along the principal component 1 (PC1) axis. Interestingly, within the treated groups, the majority of samples clustered according to age rather than BMP‐2 dose, as can be observed in PC2. There was slight separation along PC3 according to BMP‐2 dose, but this split was not as obvious as in PC1 and 2. These observations were verified by hierarchical clustering analysis (Supplemental Fig. S2), which also showed overall clustering of treated samples based on age rather than BMP‐2 dose.

**Table 1:** Summary of Gene Targets for qRT‐PCR Characterization of Harvested Tissues

Group	Genes
Osteogenic	*Runx2, Bmp2, Osx, Opg, Colla1, On, Rankl, Opn, Ocn*
Myogenic	*Pax7, Myf5, Myod1, Myog, Myh2, Ckm*
Angiogenic	*Vegfa, Epas1*
Chondrogenic	*Col2a1, Acan, Sox9, Tgfb1, Frzb*
Inflammatory	*Ifng, Tnf, Il1a, Il1b, Il6, Mcp1, Ccl3, Ccr7, Csf1*
Anti‐inflammatory	*Il1rn, Il10, Tgfb1*
Matrix remodeling	*Mmp2, Mmp3, Mmp9, Mmp13, Timp1, Adamts4, Adamts5*
Housekeeping	*Hprt1, Rplp1, Rpl13a, Gapdh, Ppia*

**Figure 2:**
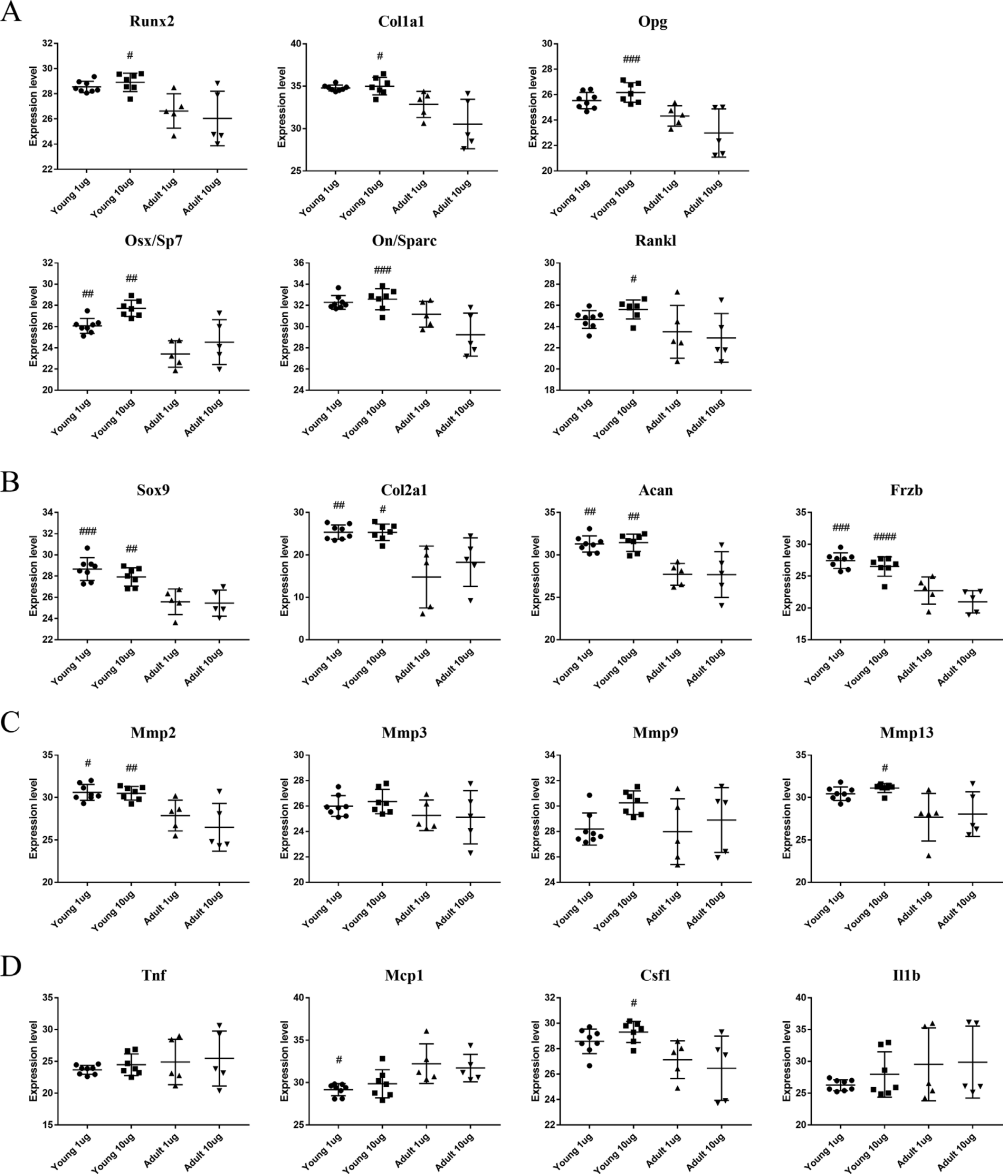
Expression differences for select genes in bone defect tissue at 1 week. Overall, young rats demonstrated elevated expression of genes relating to osteogenesis (*A*), chondrogenesis (*B*), and matrix remodeling (*C*) at 1 week, particularly at the higher 10 µg BMP‐2 dose. There were relatively few inflammatory gene differences (*D*), as the young rats exhibited lower expression of *Mcp1* but higher expression of *Csf1*. There was no significant effect of BMP‐2 dose on gene expression for either age group. Statistics: 1‐way ANOVA with Tukey's post hoc tests for multiple comparisons or the nonparametric equivalent Kruskal‐Wallis with Dunn's post hoc tests #*p *< 0.05, ##*p *< 0.01, ###*p *< 0.001, ####*p *< 0.0001 versus adult of same dose, *n* = 5–8/group.

After removing the unoperated samples from the analyses, we used ANOVA to further evaluate the contributions of age and BMP‐2 dosage to the expression of each gene. Overall, the young animals demonstrated elevated expression of genes related to osteogenesis (Fig. 2 *A*) compared with adult animals, including *Runx2, Osx/Sp7, Col1a1, Opg*, and *On/Sparc* and the osteoclastogenesis gene, *Rankl*. Similarly, the young rats also had higher expression of chondrogenic genes *Sox9, Col2a1, Acan*, and *Frzb*, as well as matrix remodeling genes *Mmp2* and *Mmp13* (Fig. 2 *B*, *C*, respectively) compared with adults. There were relatively few inflammatory (Fig. 2 *D*) or myogenic (Supplemental Fig. S3) gene expression differences: expression of *Csf1* and *Myog* were increased, whereas *Mcp1* expression was decreased in young rats. Surprisingly, differential gene expression with respect to BMP‐2 dosing was much reduced relative to the effect of age. Many of these gene expression differences were validated with a linear model approach controlling for multiple comparisons using a 5% FDR criterion (Supplemental Fig. S*4*). This analysis revealed 21 significant gene expression differences between young and adult rats (Supplemental Fig. S4*A*), of which only 1 or 2 are expected to be false positives. Similar to what was observed with ANOVA, there were very few BMP‐2 dose‐related differences (Supplemental Fig. S4*B*). Only *Osx* exhibited decreased expression at the low 1 µg dose. Although very few significant differences were attributed to BMP‐2 dose, the PCA results (separation observed in PC3) suggest that a larger sample size may have increased power sufficiently to enable detection of more dose‐dependent effects.

### Evaluation of new bone formation

**Figure 3:**
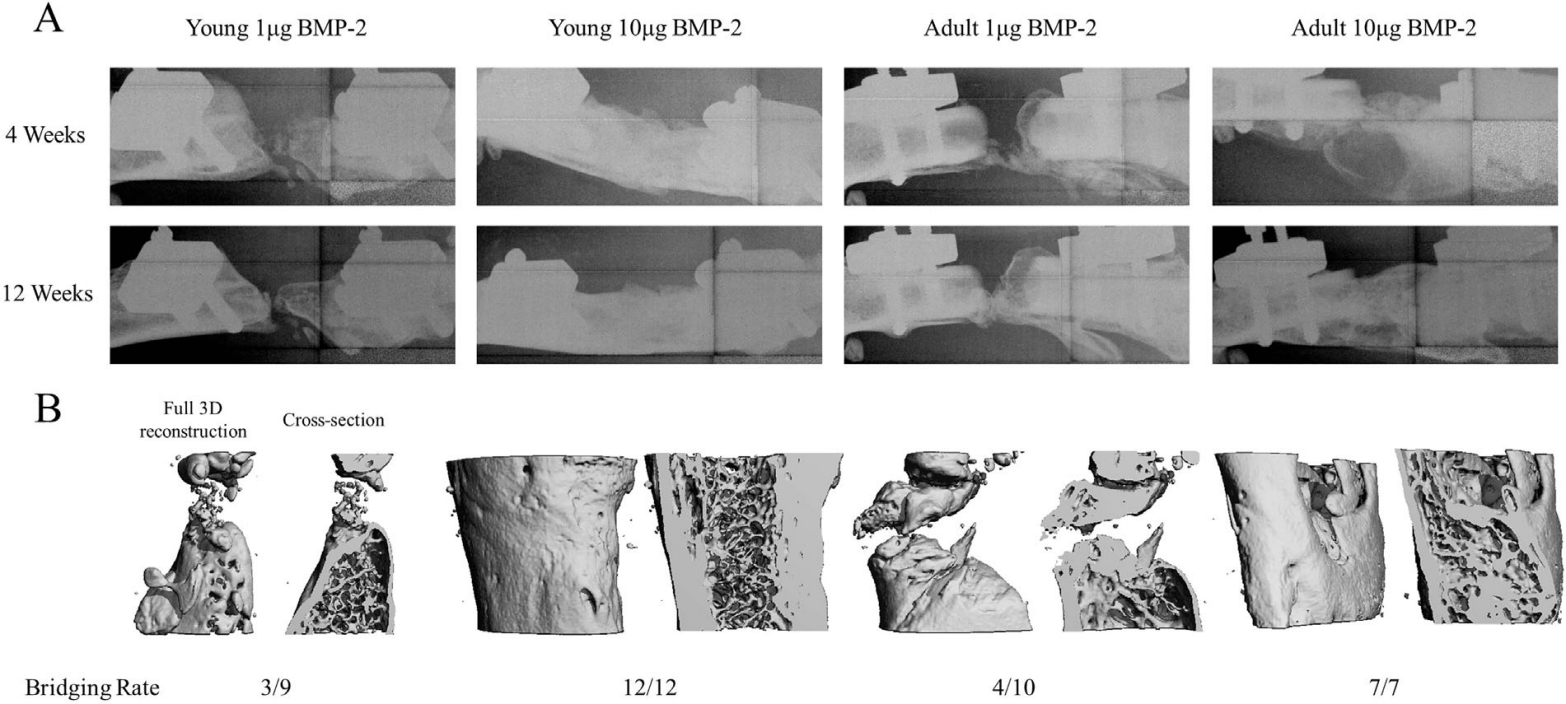
Representative radiographs and three‐dimensional µCT reconstructions of femoral bone defects. Four‐ and 12‐week radiographs (*A*) and 12‐week µCT reconstructions (*B*) demonstrated a clear effect of BMP‐2 dose on new bone formation for both age groups. The low 1 µg BMP‐2 dose resulted in poor bone regeneration with impaired defect bridging (<50% for both ages), whereas the higher 10 µg BMP‐2 dose resulted in much more robust bone formation and 100% bridging for both age groups. In addition, the morphology of the newly formed bone at the 10 µg BMP‐2 dose appeared more mature and organized from the µCT cross‐sectional view, particularly in the young rats.

**Figure 4:**
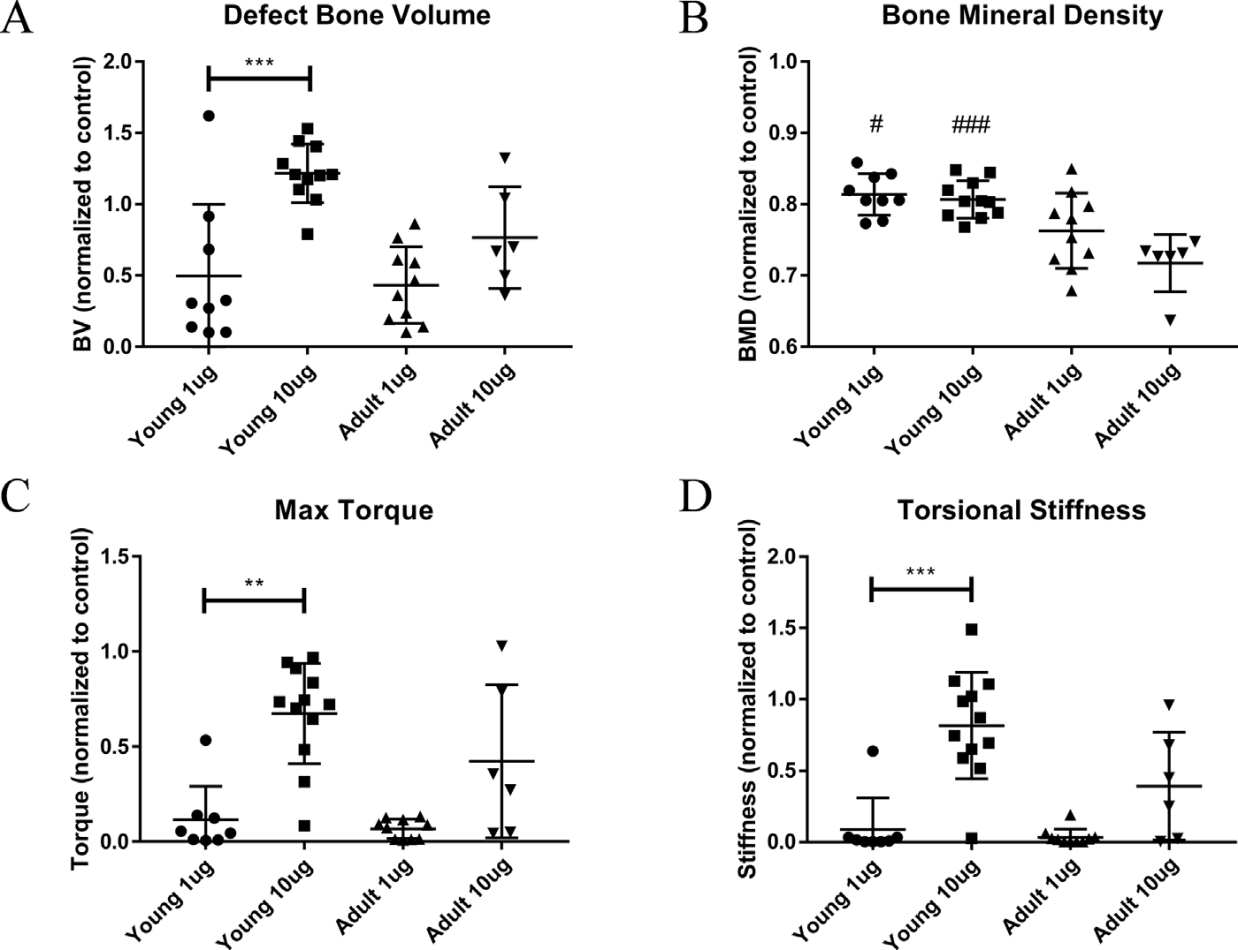
Quantitative µCT and biomechanics analysis of regenerated bone at 12 weeks. µCT analysis of defect bone volume (*A*) revealed young rats regenerated significantly more bone in the defect when BMP‐2 dose was increased from 1 to 10 µg. In contrast, the adult rats exhibited no significant increase in defect bone formation with increasing BMP‐2 dose. The regenerated bone in young rats also exhibited higher bone mineral densities (*B*) at both BMP‐2 doses compared with adult rats. Finally, torsional testing of the regenerated femurs revealed a significant dose‐dependent increase in maximum torque (*C*) and torsional stiffness (*D*) for the regenerated defects of young rats only. There were no significant differences in mechanical properties between the age groups at either dose level. Statistics: 1‐way ANOVA with Tukey's post hoc tests for multiple comparisons or the nonparametric equivalent Kruskal‐Wallis with Dunn's post hoc tests *p < 0.05, **p < 0.01, ***p < 0.001 as indicated. #*p *< 0.05, ##*p *< 0.01, ###*p *< 0.001 versus adult of same dose, *n* = 6–12/group.

Radiographical assessment at 12 weeks (Fig. 3 *A*) demonstrated that both the young and adult rats had qualitatively lower bone mineral formation at the low 1 µg BMP‐2 dose; less than 50% of defects bridged in both groups (3/9 for Young 1 µg, 4/10 for Adult 1 µg). In contrast, at the higher 10 µg BMP‐2 dose, both groups had more robust bone formation and all defects were bridged by 12 weeks (12/12 for Young 10 µg, 7/7 for Adult 10 µg). Micro‐computed tomography reconstructions (Fig. 3 *B*) supported these observations and showed a more detailed 3‐D visualization of the BMP‐2 dose effect on bone formation. Quantitative µCT analysis did not yield any differences in defect bone volume between age groups at either of the BMP‐2 doses, but the young rats demonstrated a clear BMP‐2 dosing effect in forming significantly more defect bone with the higher BMP‐2 dose, whereas the adult rats did not (Fig. 4 *A*). Furthermore, the newly formed bone in the young rats exhibited significantly higher bone mineral density at both doses compared with that of the adult rats (Fig. 4 *B*).

### Biomechanical properties of regenerated bones

Torsional testing of the harvested femurs revealed functional differences in the strengths of the regenerated bones. The maximum torque to failure (Fig. 4 *C*) and torsional stiffness (Fig. 4 *D*) were significantly increased with BMP‐2 dose only for the young animals, whereas the adult animals demonstrated no significant BMP‐2 dosing effect. Within each dose level, these mechanical parameters were not significantly different between young and adult animals. Thus, as seen with the µCT results, the young animals demonstrated a significant improvement overall, with increased BMP‐2 dosing resulting in better functional biomechanical properties.

### Histological characterization

**Figure 5:**
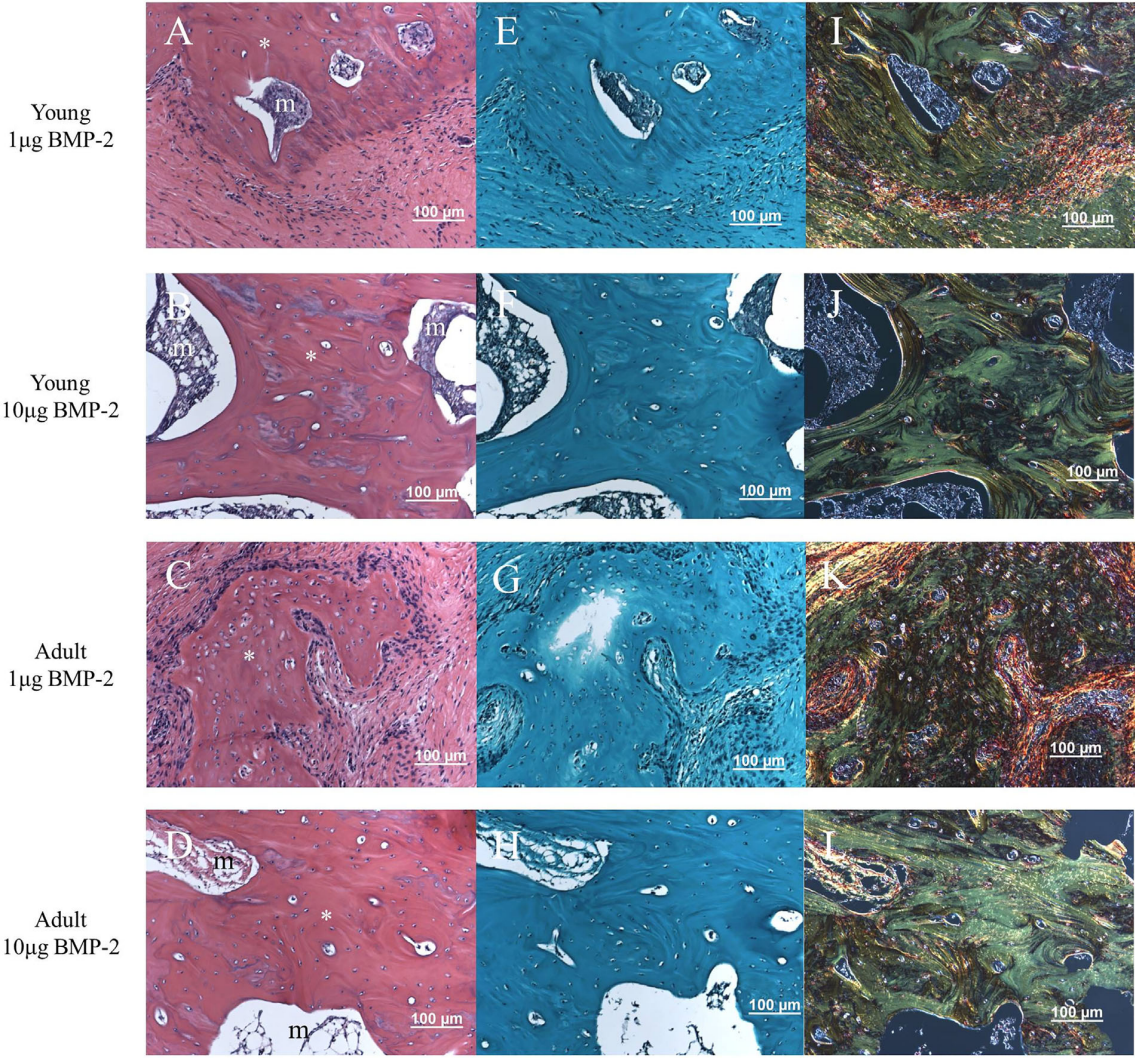
Histological staining of bone defects at 12 weeks. H&E staining (*A–D*) of representative sections from each group revealed interesting morphological differences. For both age groups, the 10 µg BMP‐2 dose samples demonstrated many large islands of new bone formation (denoted by the asterisk symbol) and more prevalent marrow‐like structures (denoted by the letter m) directly adjacent to these areas of new bone. This is in contrast to the 1 µg BMP‐2 dose samples, which demonstrated much fewer and smaller areas of new bone formation that were primarily surrounded by soft, fibrous tissue. No positive Safranin‐O staining (*E–H*) was observed within the defect space for any of the samples. Picrosirius red staining (*I–L*) of collagen fibers demonstrated that much of the new bone formed, particularly in the 10 µg BMP‐2 samples of both ages, were more mature and lamellar‐like in structure (green).

Representative samples from each treatment group were sectioned and stained with H&E, Safranin‐O and Fast Green (Saf‐O), and Picrosirius red. H&E staining (Fig. 5 *A–D*) revealed clear morphologic differences in the tissues formed within the bone defect, particularly between the low and high BMP‐2 dose samples. Areas of new bone formation were observed in all samples, but these areas were much larger and more widespread throughout the defect in the high‐dose BMP‐2 samples of both age cohorts. Furthermore, the high‐dose BMP‐2 samples exhibited formation of marrow‐like structures directly adjacent to these areas of new bone formation. In contrast, the low‐dose BMP‐2 samples demonstrated only small sparse islands of new bone formation surrounded by mostly fibrous tissue with cellular infiltrate (as indicated by the many nuclei).

Saf‐O staining (Fig. 5 *E–H*) revealed negligible cartilage present in the defect at 12 weeks post‐surgery for all groups. Finally, Picrosirius red staining (Fig. 5 *I–L*) and visualization under polarized light demonstrated that the 10 µg–treated samples in each age group had more areas of collagen fiber alignment in the areas of new bone formation, indicative of mature lamellar bone. Conversely, the 1 µg–treated samples exhibited a much more disorganized collagen structure, typical of woven bone deposition.

## Discussion

The use of BMP‐2 clinically, both for on‐label and off‐label indications, remains a highly contentious topic because of its potential for detrimental side effects. In July 2008, 6 years after the initial approval of BMP‐2 use in humans, the FDA issued a black box warning for BMP‐2 in cervical spine applications because of potential life‐threatening complications.^35^ Furthermore, a recent study noted that many of the original industry‐sponsored clinical trial publications concerning BMP‐2 safety and efficacy had underreported adverse events.^36^ To date, BMP‐2 is only approved for three indications: lumbar spine fusion, open tibial fractures, and sinus/alveolar ridge augmentations.^37^ And yet, off‐label use remains prevalent. This is despite two recent reports describing unapproved use in 66% and 79% of cases reporting adverse events to the FDA, respectively.^38^, ^39^ The continued use of BMP‐2 by clinicians in this manner suggests the lack of potential alternatives as well as the recognized benefits of BMP‐2 therapy. However, it also highlights the insufficient guidelines currently in place on how to best implement BMP‐2 therapies, and further underscores our poor fundamental understanding of the biological factors that influence BMP‐2‐mediated bone repair.

One patient population who could benefit most from improved BMP‐2 treatment strategies are pediatric (skeletally immature) patients, for whom all BMP use is currently contraindicated and yet still occurs.^25^ Presently, little is known about the influence of age on the efficacy of BMP‐2 treatment and whether dosing should be adjusted to mitigate potential adverse events. In this work, we sought to address this important problem by studying BMP‐2‐mediated healing in a preclinical large segmental bone defect model in rats. To the best of our knowledge, this work represents the first study to directly assess how young and adult rats respond to a clinically approved bone healing therapy: BMP‐2 delivered in a collagen sponge.

In support of our hypothesis, early gene expression analysis revealed that young rats demonstrated an elevated expression of many critical genes involved in the normal bone‐healing response. This partially contradicts findings from another group,^8^, ^40^ which showed that young, adult, and elderly rats all had similar overall levels of increased skeletal gene expression after femoral fracture, despite delayed union in the older cohorts. The main difference found in their study was in the temporal expression patterns: Young rats had higher levels of mRNA expression that peaked and then returned to baseline levels after 4 weeks, whereas many genes in the older animals remained upregulated up to 6 weeks after fracture,^40^ including markers of osteoblastic and osteoclastic activity.^8^ It is worth noting that these studies utilized a different bone injury rat model, as a simple closed femoral fracture with limited stabilization represents a major change in injury severity and mechanical healing environment compared with large segmental defect repair with BMP‐2 treatment and consequently may involve different signaling pathways and cellular processes.

Interestingly, in the present study, there were very few early differences in inflammatory gene expression, which is counter to our initial hypothesis. Only *Mcp1* and *Csf1* were differentially expressed between young and adult rats. Both factors are involved in macrophage recruitment and function, which suggests an altered macrophage response and supports recent work highlighting the importance of tissue resident macrophages in bone homeostasis and repair.^41^ The relative lack of inflammatory gene expression differences is more surprising considering the obvious differences observed in the physical condition of the animals. The young rats seemed to tolerate the surgery relatively well: A few demonstrated some swelling that resolved without intervention within the first 2 weeks post‐injury, but most seemed to recover with no complications. In contrast, the vast majority of adult rats exhibited more signs of prolonged distress (porphyrin staining, poor grooming, hunched posture, and weight loss) and several had to be euthanized early and excluded from the study because of declining health. Perhaps the chosen 1‐week time point was not early enough to detect the changes in local gene expression common to the acute inflammatory phase of bone healing, which has been suggested to peak within the first 72 hours post‐injury.^42^, ^43^ It is also possible that inflammatory gene biomarkers alone, despite our panel including many influential cytokines such as tumor necrosis factor, interferon gamma, and a range of interleukins, were insufficient to discern differences in inflammation. Instead, analysis of local immune cell populations or systemic immune profile characterization of serum and/or circulating cells might have been more informative for the time point chosen. In fact, it has been demonstrated that there are age‐related changes in immune cells (both number and function) recruited to sites of injury. In particular, Swift and colleagues showed that aged mice recruited higher numbers of macrophages and T cells to dermal wounds compared with young mice, and furthermore, that these aged macrophages exhibited reduced phagocytic capacity,^44^ which corroborates the diminished *Mcp1* expression exhibited by young rats observed in this study. In addition, Gibon and colleagues found that aging polarizes bone marrow macrophages toward a pro‐inflammatory M1 phenotype,^45^ which further suggests that focusing on inflammation at the cellular level may be more informative than gene expression alone.

Despite our long‐term results indicating higher BMP‐2 dose sensitivity with young rats, these did not result in more complications compared with adult rats, contrary to what one might expect, bearing in mind all the concerns with BMP‐2 use in pediatric patients. Inflammation at the terminal week 12 time point was assessed by immunohistochemistry for iNOS expression (Supplemental Fig. S5). Again, no age‐related differences were observed. However, the 1 µg BMP‐2 dose samples qualitatively seemed to have higher local iNOS expression within the bone defect compared with the 10 µg dose samples. This indicates that the defects treated with 1 µg BMP‐2 exhibited prolonged inflammation locally, which may have contributed to the poor healing observed in these samples. Beyond massive inflammation, another common adverse effect of high‐dose BMP‐2 treatment is ectopic mineralization. However, in this study, the levels of ectopic mineralization were comparable between young and adult rats (Supplemental Fig. S6), if not marginally less in the young rats: 6/12 young and 5/7 adult rats had appreciable amounts of ectopic mineralization (>5 mm^3^) at the high 10 µg BMP‐2 dose. Overall, the degree of ectopic bone was much lower than that observed in a previous study, involving a higher dose of BMP‐2.^20^ Taken together, the relatively low levels of inflammation and minimal amounts of ectopic mineralization in the young rats suggest that these potential complications can be mitigated in pediatric patients, particularly if a conservative approach is taken that utilizes lower doses of BMP‐2.

An intriguing observation from this study was that early gene expression differences (or lack thereof) was not predictive of long‐term healing outcomes. Most of the early gene expression differences were age‐dependent, whereas the long‐term functional differences in bone formation and mechanical properties were primarily dose‐dependent, particularly in the young animals. The disconnect between early and late differences is not completely unexpected because this study only characterized gene expression at a single time point (1 week post‐surgery), providing a snapshot of a highly complex and dynamic early healing environment. Moreover, quantification of mRNA transcripts can be an imperfect analog for protein levels, and this technique does not account for subsequent modulation of translational or post‐translational regulators, all of which could influence protein synthesis and activity. It is thus likely that changes to these complicated and interdependent mechanisms may have influenced the healing and inflammatory response with age and were not completely captured here.

One potential limitation of this work is the absence of a negative control group such as an untreated bone defect or treatment with collagen sponge only (no BMP‐2). However, the main focus of this study was to investigate how age and BMP‐2 dose impact the response to BMP‐2 treatment, not how BMP‐2 impacts healing compared with no treatment. Our group has also previously shown that empty bone defects or those receiving carrier alone with no BMP‐2 exhibited little to no bone formation and never achieved functional bridging.^29^, ^30^, ^46^ Furthermore, others have demonstrated that delivery of collagen sponge loaded with saline to critical size defects results in minimal bone formation and very low expression of osteogenic and angiogenic genes compared with BMP‐2 treatment.^47^ Consequently, we excluded untreated or collagen sponge only control groups given that they would provide limited new insight and prioritized assessment of the interactive effects of age and dose on BMP‐2 treatment response.

Another limitation of this study is the lack of BMP‐2 release characterization. Although release of BMP‐2 from collagen sponge was not directly assessed in this study, we have previously characterized BMP‐2 release profiles from collagen sponge, both in vivo^29^ and in vitro.^20^ In those studies, we tested multiple doses of BMP‐2 and observed similar release kinetics. However, it remains to be seen if there are age‐related changes to BMP‐2 release from collagen sponge in vivo. The increased MMP gene expression observed in young rats suggests that young rats may be able to remodel/degrade collagen more rapidly, leading to faster BMP‐2 release. An additional experiment, perhaps involving delivery of fluorescently tagged BMP‐2, would be needed to address whether age impacts the in vivo BMP‐2 release profile from collagen sponge. More extensive studies are also needed to better understand the less robust bone‐healing response exhibited by adult animals overall. Other groups have demonstrated that aging reduces fracture callus vascularization,^48^ decreases callus volume and cartilage/bone content,^49^ impairs osteogenic differentiation of bone marrow cells,^50^ and diminishes periosteal stem cell populations.^51^ Further investigations into these aging‐related deficits and how they are impacted by treatments such as BMP‐2 may reveal alternative approaches for optimizing current therapeutics as well as uncover potential targets for new therapies.

In summary, the work presented here represents the first investigation into the role of age on BMP‐2‐mediated segmental bone defect repair using the current clinical standard, BMP‐2 delivered in collagen sponge. We found that young rats exhibited elevated early expression of genes involved in bone healing and were able to form better‐quality bone for long‐term phase. Of clinical significance, our results revealed a clear effect of age on BMP‐2 dose sensitivity as young rats appeared more responsive to increases in dosing than adult rats, without experiencing a higher complication rate. These results suggest that adjusting BMP‐2 dose based on age may be an advantageous strategy to help minimize the potential risks associated with BMP therapy.

## Disclosures

All authors state that they have no conflicts of interest.

## Supporting information

Supporting Figures S1.

## References

[1] Oryan A , Alidadi S , Moshiri A , Maffulli N. Bone regenerative medicine: classic options, novel strategies, and future directions. J Orthop Surg Res. 2014;9(1):18. 24628910 10.1186/1749-799X-9-18 PMC3995444

[2] Mehta M , Schmidt‐Bleek K , Duda GN , Mooney DJ. Biomaterial delivery of morphogens to mimic the natural healing cascade in bone. Adv Drug Deliv Rev. 2012;64(12):1257–76. 22626978 10.1016/j.addr.2012.05.006 PMC3425736

[3] Jones CB , Sabatino CT , Badura JM , Sietsema DL , Marotta JS. Improved healing efficacy in canine ulnar segmental defects with increasing recombinant human bone morphogenetic protein‐2/allograft ratios. J Orthop Trauma. 2008;22(8):550–9. 18758287 10.1097/BOT.0b013e318180f0f0

[4] Krishnan L , Priddy LB , Esancy C , et al. Hydrogel‐based delivery of rhBMP‐2 improves healing of large bone defects compared with autograft. Clin Orthop Relat Res, 2015;473(9):2885–97. 25917422 10.1007/s11999-015-4312-z PMC4523508

[5] Birkhold AI , Razi H , Duda GB , Weinkamer R , Checa S , Willie BM. The influence of age on adaptive bone formation and bone resorption. Biomaterials. 2014;35(34):9290–301. 25128376 10.1016/j.biomaterials.2014.07.051

[6] Lu C , Miclau T , Hu D , et al. Cellular basis for age‐related changes in fracture repair. J Orthop Res. 2005;23(6):1300–7. 15936915 10.1016/j.orthres.2005.04.003.1100230610 PMC2844440

[7] Mehta M , Strube P , Peters A , et al. Influences of age and mechanical stability on volume, microstructure, and mineralization of the fracture callus during bone healing: is osteoclast activity the key to age‐related impaired healing? Bone. 2010;47(2):219–28. 20510391 10.1016/j.bone.2010.05.029

[8] Meyer RA Jr, Desai BR , Heiner DE , Fiechtl J , Porter S , Meyer MH. Young, adult, and old rats have similar changes in mRNA expression of many skeletal genes after fracture despite delayed healing with age. J Orthop Res. 2006;24(10):1933–44. 16894589 10.1002/jor.20124

[9] Strube P , Sentuerk U , Riha T , et al. Influence of age and mechanical stability on bone defect healing: age reverses mechanical effects. Bone. 2008;42(4):758–64. 18280233 10.1016/j.bone.2007.12.223

[10] Ekeland A , Engesoeter LB , Langeland N. Influence of age on mechanical properties of healing fractures and intact bones in rats. Acta Orthop Scand. 1982;53(4):527–34. 7102268 10.3109/17453678208992252

[11] Gaston MS , Simpson AH. Inhibition of fracture healing. J Bone Joint Surg Br. 2007;89(12):1553–60. 18057352 10.1302/0301-620X.89B12.19671

[12] Gruber R , Koch H , Doll BA , Tegtmeier F , Einhorn TA , Hollinger JO. Fracture healing in the elderly patient. Exp Gerontol. 2006;41(11):1080–93. 17092679 10.1016/j.exger.2006.09.008

[13] Lindaman LM. Bone healing in children. Clin Podiatr Med Surg. 2001;18(1):97–108. 11344982

[14] Osyczka AM , Damek‐Poprawa M , Wojtowicz A , Akintoye SO. Age and skeletal sites affect BMP‐2 responsiveness of human bone marrow stromal cells. Connect Tissue Res. 2009;50(4):270–7. 19637063 10.1080/03008200902846262 PMC2905683

[15] Lee KB , Taghavi CE , Hsu MS , et al. The efficacy of rhBMP‐2 versus autograft for posterolateral lumbar spine fusion in elderly patients. Eur Spine J. 2010;19(6):924–30. 20041271 10.1007/s00586-009-1248-6 PMC2899988

[16] Yamaji K , Kawanami M , Matsumoto A , et al. Effects of dose of recombinant human BMP‐2 on bone formation at palatal sites in young and old rats. Dent Mater J. 2007;26(4):481–6. 17886450 10.4012/dmj.26.481

[17] Cahill KS , et al. Prevalence, complications, and hospital charges associated with use of bone‐morphogenetic proteins in spinal fusion procedures. JAMA. 2009;302(1):58–66. 19567440 10.1001/jama.2009.956

[18] Cahill KS , McCormick PC , Levi AD. A comprehensive assessment of the risk of bone morphogenetic protein use in spinal fusion surgery and postoperative cancer diagnosis. J Neurosurg Spine. 2015;23(1):86–93. 25860517 10.3171/2014.10.SPINE14338

[19] Epstein NE. Complications due to the use of BMP/INFUSE in spine surgery: the evidence continues to mount. Surg Neurol Int. 2013;4(Suppl 5):S343–52. 23878769 10.4103/2152-7806.114813 PMC3717531

[20] Krishnan L , Priddy LB , Esancy C , et al. Delivery vehicle effects on bone regeneration and heterotopic ossification induced by high dose BMP‐2. Acta Biomater. 2017;49:101–112. 27940197 10.1016/j.actbio.2016.12.012 PMC5253230

[21] Zara JN , Siu RK , Zhang X , et al. High doses of bone morphogenetic protein 2 induce structurally abnormal bone and inflammation in vivo. Tissue Eng Part A. 2011;17 (9–10):1389–99. 21247344 10.1089/ten.tea.2010.0555 PMC3079169

[22] Ritting AW , Weber EW , Lee MC. Exaggerated inflammatory response and bony resorption from BMP‐2 use in a pediatric forearm nonunion. J Hand Surg Am. 2012;37(2):316–21. 22119603 10.1016/j.jhsa.2011.10.007

[23] MacDonald KM , Swanstrom MM , McCarthy JJ , Nemeth BA , Guliani TA , Noonan KJ. Exaggerated inflammatory response after use of recombinant bone morphogenetic protein in recurrent unicameral bone cysts. J Pediatr Orthop. 2010;30(2):199–205. 20179570 10.1097/BPO.0b013e3181cec35b

[24] Oetgen ME , Richards BS. Complications associated with the use of bone morphogenetic protein in pediatric patients. J Pediatr Orthop. 2010;30(2):192–8. 20179569 10.1097/BPO.0b013e3181d075ab

[25] Papanna MC , Saldanha KA , Kurian B , Fernandes JA , Jones S. The use of recombinant morphogenic protein‐2(rhBMP‐2) in children undergoing revision surgery for persistent non‐union. Strategies Trauma Limb Reconstr. 2016;11(1):53–8. 26984411 10.1007/s11751-016-0251-9 PMC4814389

[26] Molinari RW , Molinari C. The use of bone morphogenetic protein in pediatric cervical spine fusion surgery: case reports and review of the literature. Global Spine J. 2016;6(1):e41–6. 26835215 10.1055/s-0035-1555660 PMC4733381

[27] Mladenov KV , Kunkel P , Stuecker R. The use of recombinant human BMP‐2 as a salvage procedure in the pediatric spine: a report on 3 cases. Eur Spine J. 2010;19 Suppl 2:S135–9. 19876660 10.1007/s00586-009-1179-2 PMC2899647

[28] Oest ME , Dupont KM , Kong HJ , Mooney DJ , Guldberg RE. Quantitative assessment of scaffold and growth factor‐mediated repair of critically sized bone defects. J Orthop Res. 2007;25(7):941–50. 17415756 10.1002/jor.20372

[29] Boerckel JD , Kolambkar YM , Dupont KM , et al. Effects of protein dose and delivery system on BMP‐mediated bone regeneration. Biomaterials. 2011;32(22):5241–51. 21507479 10.1016/j.biomaterials.2011.03.063 PMC3129848

[30] Kolambkar YM , Dupont KM , Boerckel JD , et al. An alginate‐based hybrid system for growth factor delivery in the functional repair of large bone defects. Biomaterials. 2011;32(1):65–74. 20864165 10.1016/j.biomaterials.2010.08.074 PMC3013370

[31] Spurgeon SL , Jones RC , Ramakrishnan R. High throughput gene expression measurement with real time PCR in a microfluidic dynamic array. PLoS One. 2008;3(2):e1662. 18301740 10.1371/journal.pone.0001662 PMC2244704

[32] Vandesompele J , De Preter K , Pattyn F , et al. Accurate normalization of real‐time quantitative RT‐PCR data by geometric averaging of multiple internal control genes. Genome Biol. 2002;3(7):RESEARCH0034. 12184808 10.1186/gb-2002-3-7-research0034 PMC126239

[33] Wolfinger RD , Gibson G , Wolfinger ED , et al. Assessing gene significance from cDNA microarray expression data via mixed models. J Comput Biol. 2001;8(6):625–37. 11747616 10.1089/106652701753307520

[34] Hettiaratchi MH , Chou C , Servies N , et al. Competitive protein binding influences heparin‐based modulation of spatial growth factor delivery for bone regeneration. Tissue Eng Part A. 2017;23(13–14):683–95. 28338419 10.1089/ten.tea.2016.0507 PMC5549832

[35] James AW , LaChaud G , Shen J , et al. A review of the clinical side effects of bone morphogenetic protein‐2. Tissue Eng Part B Rev. 2016;22(4):284–97. 26857241 10.1089/ten.teb.2015.0357 PMC4964756

[36] Carragee EJ , Hurwitz EL , Weiner BK. A critical review of recombinant human bone morphogenetic protein‐2 trials in spinal surgery: emerging safety concerns and lessons learned. Spine J. 2011;11(6):471–91. 21729796 10.1016/j.spinee.2011.04.023

[37] McKay WF , Peckham SM , Badura JM. A comprehensive clinical review of recombinant human bone morphogenetic protein‐2 (INFUSE Bone Graft). Int Orthop. 2007;31(6):729–34. 17639384 10.1007/s00264-007-0418-6 PMC2266665

[38] Woo EJ. Adverse events reported after the use of recombinant human bone morphogenetic protein 2. J Oral Maxillofac Surg. 2012;70(4):765–7. 22177811 10.1016/j.joms.2011.09.008

[39] Woo EJ. Adverse events after recombinant human BMP2 in nonspinal orthopaedic procedures. Clin Orthop Relat Res. 2013;471(5):1707–11. 23132207 10.1007/s11999-012-2684-x PMC3613534

[40] Desai BJ , Meyer MH , Porter S , Kellam JF , Meyer RA Jr. The effect of age on gene expression in adult and juvenile rats following femoral fracture. J Orthop Trauma. 2003;17(10):689–98. 14600568 10.1097/00005131-200311000-00005

[41] Sinder BP , Pettit AR , McCauley LK. Macrophages: their emerging roles in bone. J Bone Miner Res. 2015;30(12):2140–9. 26531055 10.1002/jbmr.2735 PMC4876707

[42] Rundle CH, et al. Microarray analysis of gene expression during the inflammation and endochondral bone formation stages of rat femur fracture repair. Bone. 2006;38(4):521–9. 16321582 10.1016/j.bone.2005.09.015

[43] Wang X , Yu YY , Lieu S , et al. MMP9 regulates the cellular response to inflammation after skeletal injury. Bone. 2013;52(1):111–9. 23010105 10.1016/j.bone.2012.09.018 PMC3513654

[44] Swift ME , Burns AL , Gray KL , DiPietro LA. Age‐related alterations in the inflammatory response to dermal injury. J Invest Dermatol. 2001;117(5):1027–35. 11710909 10.1046/j.0022-202x.2001.01539.x

[45] Gibon E , Loi F , Cordova LA , et al. Aging affects bone marrow macrophage polarization: relevance to bone healing. Regen Eng Transl Med. 2016;2(2):98–104. 28138512 10.1007/s40883-016-0016-5 PMC5270653

[46] Dupont KM , Sharma K , Stevens HY , Boerckel JD , Garcia AJ , Guldberg RE. Human stem cell delivery for treatment of large segmental bone defects. Proc Natl Acad Sci U S A. 2010;107(8):3305–10. 20133731 10.1073/pnas.0905444107 PMC2840521

[47] Hussein KA , Zakhary IE , Elawady AR , et al. Difference in soft tissue response between immediate and delayed delivery suggests a new mechanism for recombinant human bone morphogenetic protein 2 action in large segmental bone defects. Tissue Eng Part A. 2012;18(5–6):665–75. 21981405 10.1089/ten.TEA.2011.0148

[48] Lu C , Hansen E , Sapozhnikova A , Hu D , Miclau T , Marcucio RS. Effect of age on vascularization during fracture repair. J Orthop Res. 2008;26(10):1384–9. 18464248 10.1002/jor.20667 PMC2846969

[49] Lopas LA , Belkin NS , Mutyaba PL , Gray CF , Hankenson KD , Ahn J. Fractures in geriatric mice show decreased callus expansion and bone volume. Clin Orthop Relat Res. 2014;472(11):3523–32. 25106797 10.1007/s11999-014-3829-x PMC4182401

[50] Nishida S , Endo N , Yamagiwa H , Tanizawa T , Takahashi HE. Number of osteoprogenitor cells in human bone marrow markedly decreases after skeletal maturation. J Bone Miner Metab. 1999;17(3):171–7. 10757676 10.1007/s007740050081

[51] Fan W , Crawford R , Xiao Y. Structural and cellular differences between metaphyseal and diaphyseal periosteum in different aged rats. Bone. 2008;42(1):81–9. 17962095 10.1016/j.bone.2007.08.048

