# Supplementary material for: The Effects of Age and Dose on Gene Expression and Segmental Bone Defect Repair After BMP‐2 Delivery
Source: JBMR Plus. 2018 Jul 16;3(2):e10068. doi: 10.1002/jbm4.10068 (PMC6383700; doi:10.1002/jbm4.10068)
Supplement: Supplementary file 1 — Supporting Figures S1. [file JBM4-3-na-s001.docx]

**Supplemental Figures**

**
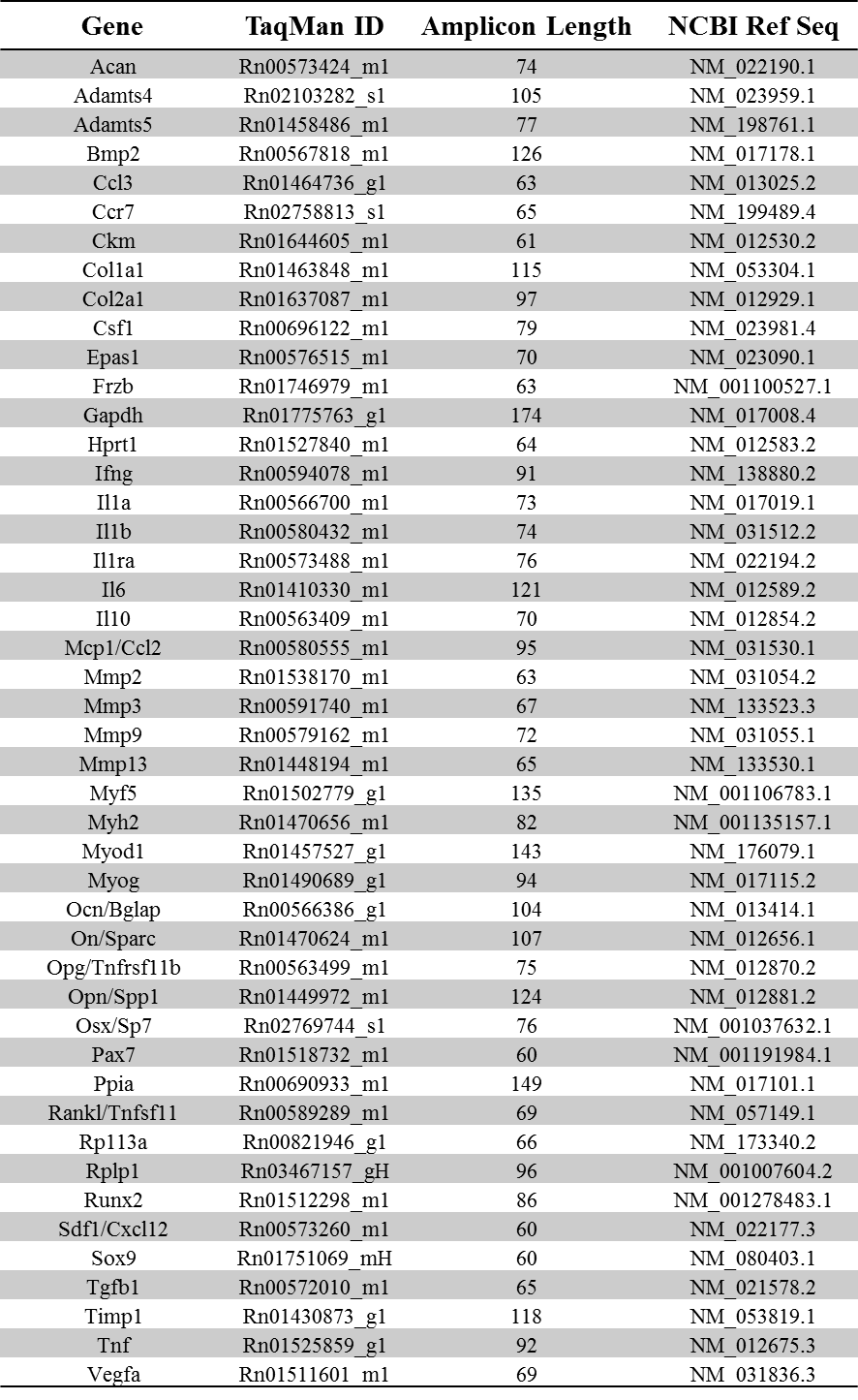
**

**Figure S1. TaqMan probes used for Fluidigm-based qRT-PCR.**

**
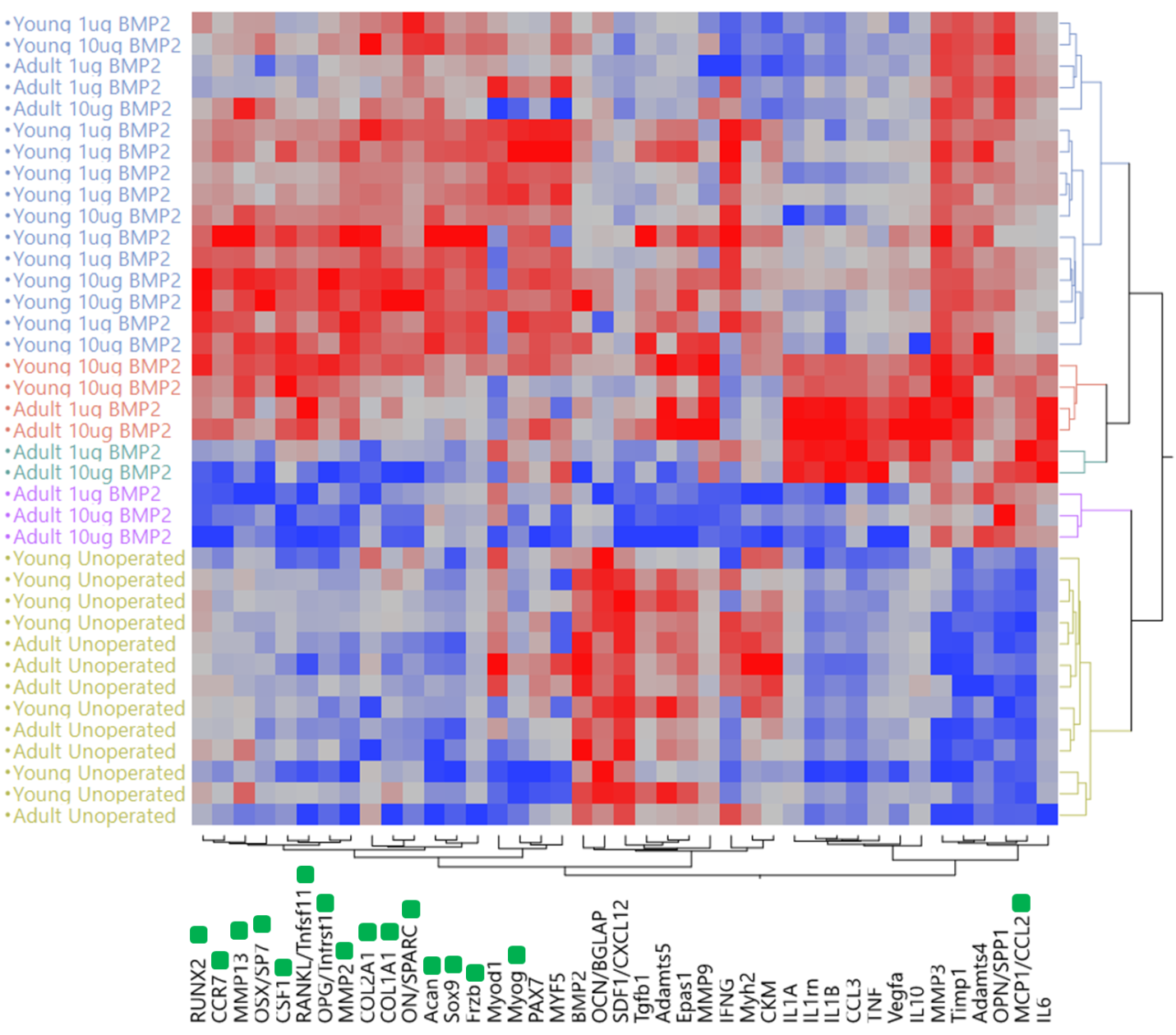
**

**Figure S2. Hierarchical clustering of bone defect samples at 1 week.** Heatmap revealed gene expression clustering primarily according to age, rather than BMP-2 dose, as well as clear separation of unoperated controls. Expression levels are represented on a scale of lower (blue) to higher (red) relative expression. Green squares denote significant difference (p<0.05) in expression of corresponding gene between young and adult animals at one or both BMP-2 doses (excluding unoperated controls).


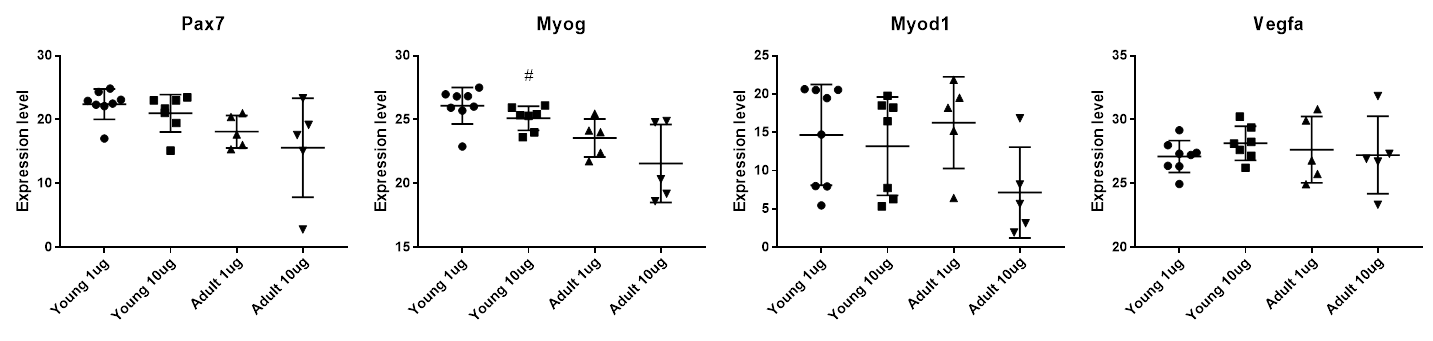


**Figure S3. Myogenic and angiogenic gene expression in bone defect tissue at 1 week.** Young rats showed elevated expression of *Myog* compared to adult rats at the 10 μg BMP-2 dose.


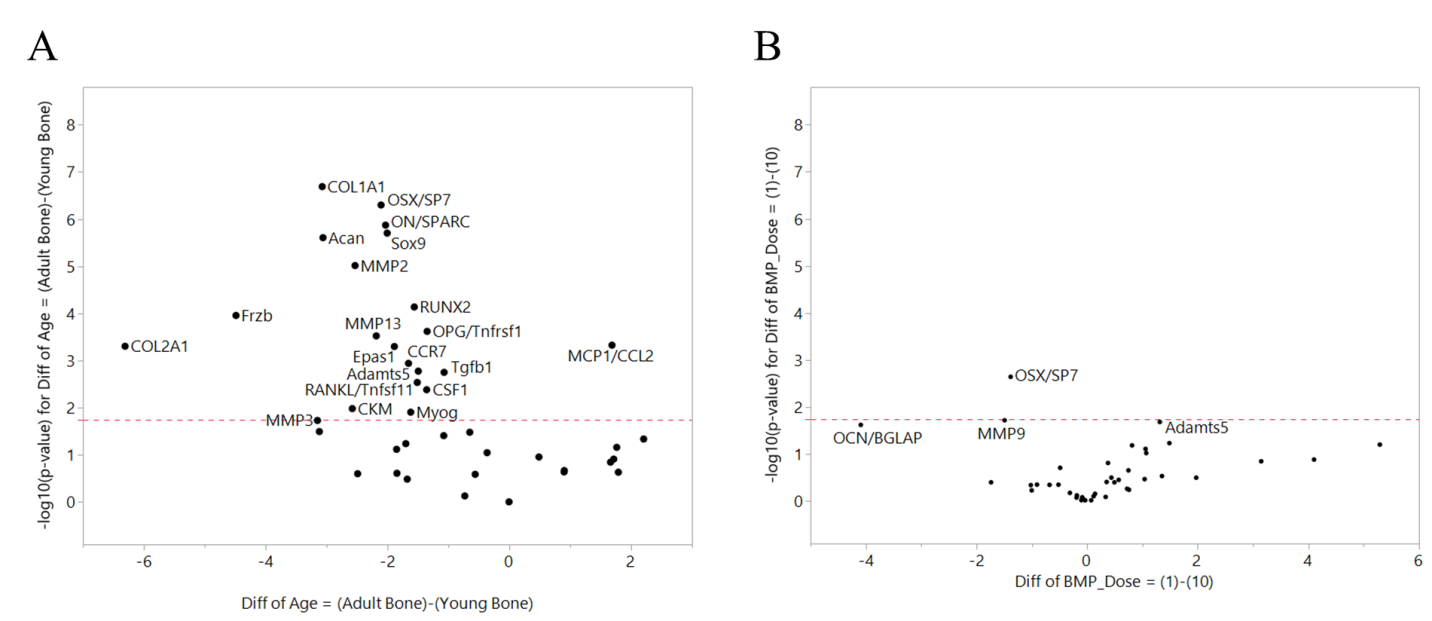


**Figure S4. Significant gene expression differences at 1 week using linear model approach with FDR.** There were 21 differentially expressed genes between young and adult animals (A), many of these overlap with those identified by ANOVA in Figure 2. In contrast, there was only 1 differentially expressed gene between the two BMP-2 doses (B): *Osx* expression was significantly lower in the samples that received 1 μg BMP-2 compared to 10 μg. All genes that are above the dashed red line are significant at a false discovery rate (FDR) of 0.05.


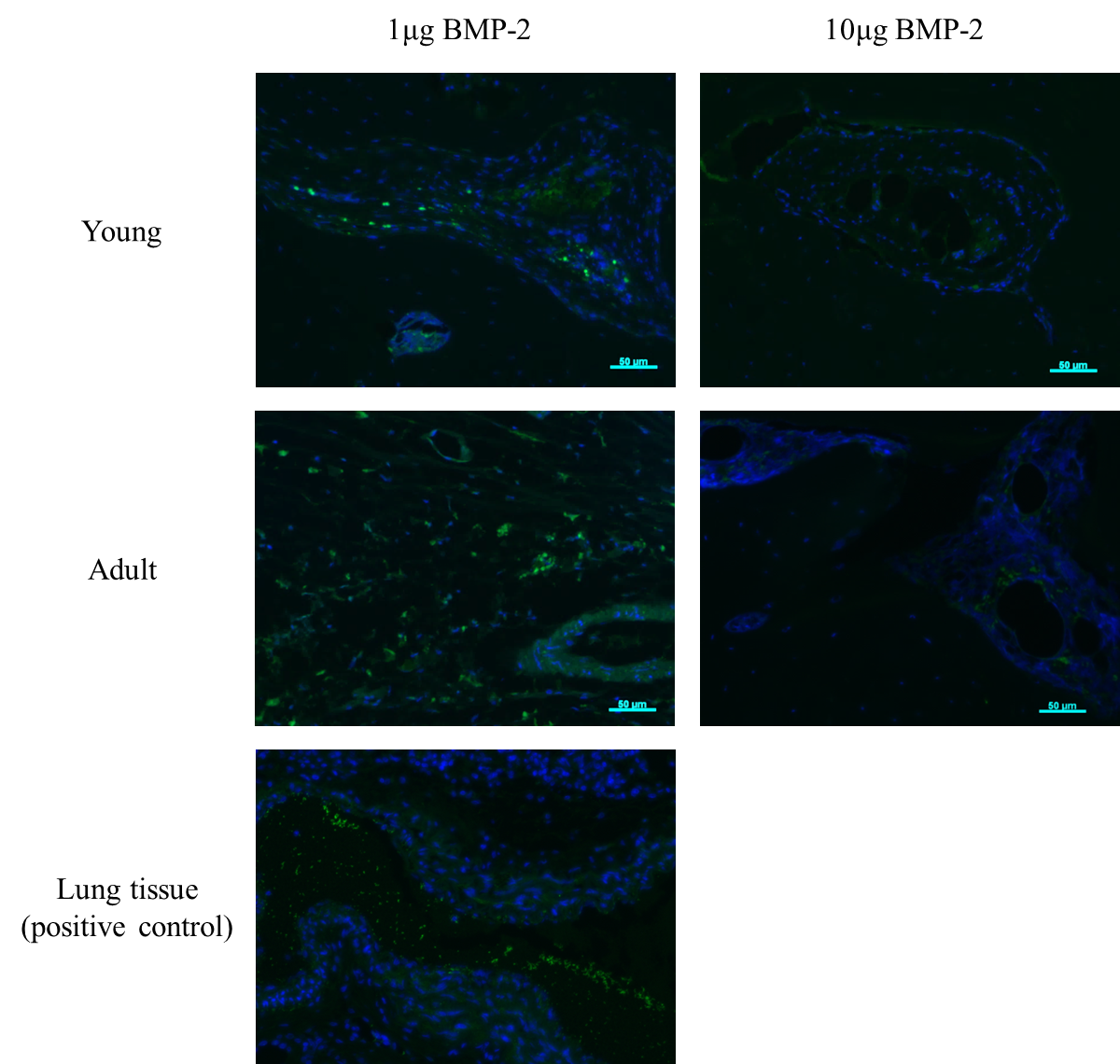


**Figure S5. Elevated iNOS expression in bone defects treated with 1μg dose of BMP-2.** Immunohistochemistry revealed qualitative differences in inducible nitric oxide synthase (iNOS) expression within the regenerated bone defects at 12 weeks post-treatment (pictures taken mid-defect). For both young and adult rats, positive iNOS staining appeared more prevalent at the 1 μg BMP-2 dose compared to the 10 μg dose. Positive iNOS staining between age groups were comparable.

**Figure S6. Quantification of ectopic mineralization at 12 weeks.** μCT analysis revealed an increase in the number of samples demonstrating appreciable amounts of ectopic bone formation (>5mm^3^) in both young and adult animals, although this increase was not statistically significant. There were no differences in amount of ectopic bone between young and adult animals at either of the BMP-2 doses tested.
